# Optical Diffraction Tomography and Raman Confocal Microscopy for the Investigation of Vacuoles Associated with Cancer Senescent Engulfing Cells

**DOI:** 10.3390/bios13110973

**Published:** 2023-11-07

**Authors:** Silvia Ghislanzoni, Jeon Woong Kang, Arianna Bresci, Andrea Masella, Koseki J. Kobayashi-Kirschvink, Dario Polli, Italia Bongarzone, Peter T. C. So

**Affiliations:** 1 Department of Diagnostic Innovation, Fondazione IRCCS Istituto Nazionale dei Tumori, Via Giacomo Venezian 1, 20133 Milan, Italy; italia.bongarzone@istitutotumori.mi.it; 2 Laser Biomedical Research Center, G. R. Harrison Spectroscopy Laboratory, Massachusetts Institute of Technology, Cambridge, MA 02139, USA; arianna.bresci@polimi.it (A.B.); kkobayas@broadinstitute.org (K.J.K.-K.); ptso@mit.edu (P.T.C.S.); 3 Department of Physics, Politecnico di Milano, Piazza L. da Vinci 32, 20133 Milan, Italy; dario.polli@polimi.it; 4 Datrix S.p.A., Foro Buonaparte 71, 20121 Milan, Italy; andrea.masella@datrixgroup.com; 5 Klarman Cell Observatory, Broad Institute of MIT and Harvard, Cambridge, MA 02142, USA; 6 CNR Institute for Photonics and Nanotechnologies (IFN), Piazza L. da Vinci 32, 20133 Milan, Italy

**Keywords:** microscopy, optical diffraction tomography, Raman spectroscopy, therapy-induced senescence, cell engulfing, vacuole, cancer, cell in cell

## Abstract

Wild-type p53 cancer therapy-induced senescent cells frequently engulf and degrade neighboring ones inside a massive vacuole in their cytoplasm. After clearance of the internalized cell, the vacuole persists, seemingly empty, for several hours. Despite large vacuoles being associated with cell death, this process is known to confer a survival advantage to cancer engulfing cells, leading to therapy resistance and tumor relapse. Previous attempts to resolve the vacuolar structure and visualize their content using dyes were unsatisfying for lack of known targets and ineffective dye penetration and/or retention. Here, we overcame this problem by applying optical diffraction tomography and Raman spectroscopy to MCF7 doxorubicin-induced engulfing cells. We demonstrated a real ability of cell tomography and Raman to phenotype complex microstructures, such as cell-in-cells and vacuoles, and detect chemical species in extremely low concentrations within live cells in a completely label-free fashion. We show that vacuoles had a density indistinguishable to the medium, but were not empty, instead contained diluted cell-derived macromolecules, and we could discern vacuoles from medium and cells using their Raman fingerprint. Our approach is useful for the noninvasive investigation of senescent engulfing (and other peculiar) cells in unperturbed conditions, crucial for a better understanding of complex biological processes.

## 1. Introduction

*TP53* wild-type human breast tumors rarely undergo pathological complete response following anticancer therapy [1,2], resulting in poor survival [3]. Their unresponsiveness to treatment has been mainly attributed to the proneness of tumor cells with normal p53 to avoid cell death by undergoing senescence [4,5], a dormant but metabolically active state. Senescent cells can persist for a long time and secrete inflammatory factors that can drive relapse by promoting survival, proliferation, and stemness [4,6,7]. Thus, understanding how senescent cancer cells can survive for long periods after treatment is paramount for developing more effective anticancer therapies. Recently, Tonnessen-Murray and colleagues showed that *TP53* wild-type breast cancer senescent cells engulf and break down vital, neighboring ones with remarkable frequency. This process confers a survival advantage to engulfing cells, as it provides nutrients and building blocks necessary for survival, explaining in part how they can persist and maintain high metabolism rates under such unfavorable conditions [8]. Notably, cell-in-cell structures derived from cell engulfment can be observed in patient tissues of several tumor types after chemotherapy and are associated with negative prognosis [9,10,11]. As this phenomenon is not yet fully understood, the efforts made towards the development of effective strategies to eliminate cancer engulfing cells have yielded unsatisfying results so far. A peculiarity of this senescence-associated engulfing process is the formation of a big vacuole within engulfing cells. This structure is necessary for the degradation of the internalized cells [11], but it persists over the span of several hours after their clearance, and it appears empty both in brightfield mode and following staining with several fluorescent markers. While small, transient vacuolar organelles such as lysosomes are crucial for cell survival, extreme vacuolization and large vacuoles are normally associated with cell death in mammalians [12,13]; thus, it is uncanny how cancer senescent cells can persevere in hostile conditions with a seemingly empty vacuole occupying most of their cytoplasm. Yet, despite the great deal of interest in cell-in-cell structures in the recent cancer literature, very little attention has been placed on vacuoles. As they constitute a major feature of tumor senescence-related engulfing, their description, including information about their chemical and morphological composition, is essential for the development of effective methods for the prevention and/or elimination of these aggressive senescent cells. In turn, this would foster more efficient, targeted anticancer strategies for (breast) tumors that are resistant to conventional treatment.

Cell-in-cell and vacuolated structures are three-dimensional (3D), architecturally complex systems. The 3D reconstruction of a biological specimen and the visualization of the molecular species that compose it are often obtained with fluorescent confocal microscopy [14,15], but in order to attain satisfying, reliable results with this technique, one needs to know all the potential, labelable targets and the specimen should efficiently absorb and retain the set of selected fluorescent dyes [16]. As the vacuolar content after clearance of the engulfed cells is still undefined and the vacuolar membrane may represent a further obstacle to dye penetration, in addition to the surrounding engulfing cell one, neither of these conditions is satisfied. Moreover, the use of dyes causes the perturbation of the biological model, which often leads to artifacts and signals that do not proportionally and/or uniquely relate to the targeted feature [16]. To overcome these problems, we employed optical diffraction tomography (ODT) and confocal Raman microscopy for label-free investigation of morphological and chemical information, respectively.

ODT is an interferometric technique that measures the refractive index (RI) of transparent samples, such as cells and subcellular structures [17,18,19,20]. The RI value of an item is linearly proportional to its mass density [21,22,23], making ODT particularly suited to probe the hollowness of engulfing-derived vacuoles, and, as the measure is label-free, it avoids typical dye-related issues [14,17,24,25]. Moreover, ODT measures the 3D RI distribution of a specimen, delivering 3D reconstructions of complex biological structures featuring sub-micrometer spatial resolution [14]. However, ODT lacks the molecular specificity necessary to interpret the biochemical content of vacuoles [14]. The solution to this problem comes from Raman spectroscopy. Raman spectroscopy uses the vibrational motions of chemical bonds as a contrast mechanism, revealing the biomolecular composition of the sample in a label-free manner, with subcellular spatial resolution (i.e., micrometer-scale resolution). This provides an endogenous signature that can be used as a fingerprint for the unique characterization of a specimen chemical content in terms of lipids, proteins, nucleic acids, carbohydrate concentration, and many others [26,27,28,29,30,31]. Our custom-build high-speed confocal Raman microscope has been successfully used for various applications including malaria diagnosis [32] and monitoring cell–drug interaction on multiple myeloma cells [33] and UV-induced cell damage [34]. Specifically, the combined Raman and quantitative-phase microscopy system demonstrated fast morphological screening of a large number of RBCs followed by chemical confirmation using Raman mapping [17], showing the powerful combination of two label-free imaging techniques. By applying a combination of ODT and confocal Raman microscopy to MCF7 *TP53* wild-type, doxorubicin (Doxo)-induced senescent engulfing cells, we were able to visualize the 3D structure of live engulfing cells, including vacuoles, and investigate vacuolar content in a completely label-free fashion, phenotyping these treacherous cells in unperturbed samples.

## 2. Materials and Methods

### 2.1. Cell Culture and Treatment

*TP53* wild-type human breast adenocarcinoma MCF7 cells were purchased from American Type Culture Collection (ATCC, VA, USA; ATCC number: HB-8065) and maintained in RPMI 1640 Medium (Gibco) supplemented with 10% fetal bovine serum (FBS, Gibco). Cells were seeded on 35 mm petri dishes with a 12 mm diameter quartz coverslip in the center (SFS-S-D12, WakenBTech, Kyoto, Japan) at a density of 50,000 cells/mL and treated with 250 nM doxorubicin (Doxo, Sigma-Aldrich, Darmstadt, Germany) 24 h after seeding to induce senescence and its related engulfing phenotype.

### 2.2. β-Galactosidase Staining

Live control and treated cells were incubated at 37 °C and 5% CO_2_ with 1 μM BioTracker 519 Green β-Gal dye (Merck) and with a 1 mM DAPI ready-to-use solution (Thermo Fisher, Waltham, MA, USA) for 15 min. Then, they were gently washed with PBS and observed in PBS under the confocal microscope Leica TCS SP8 X (Leica Microsystems, Wetzlar, Germany).

### 2.3. Optical Diffraction Tomography

MCF7 cells were cultured and treated as described in Section 2.1. At 18, 24, 48, and 72 h after treatment, engulfing cells were either fixed with 4% paraformaldehyde (PFA, Sigma) or observed and imaged live with the commercial microscope HT-1H (Tomocube, Daejeon, Republic of Korea). Images of the 3D RI distribution of samples were rendered using Tomostudio software (Tomocube, Republic of Korea).

### 2.4. Raman Spectroscopy

This study employs a home-built multimodal microscope including multi-position and multi-timepoint fluorescence imaging and point-scanning Raman microscopy [35]. A 785 nm continuous-wave Ti:Sapphire laser coupled to a 532 nm pump laser was used as an excitation source. The source is integrated in an Olympus IX83 fluorescence inverted microscope body. White light illumination is coupled for both bright-field and fluorescence imaging. The fluorescence and Raman imaging modalities are switched by swapping dichroic filters with auto-turrets. Light is focused on the sample via an Olympus UPLSAPO 60X 1.2-NA water immersion objective. The backscattered light is collected using the same objective. The system includes both a galvo mirror-based scanning and stage scanning in order to acquire each field of view (FOV) and multiple FOVs, respectively, delivering high-throughput Raman measurements. Through a 785 nm dichroic mirror, brightfield and fluorescence signals are short-pass filtered from the Raman signal and sent to a spectrograph (Holospec f/1.8i 785 nm model). A Princeton Instruments PIXIS 100BR eXcelon measures the Raman signal. An Orca Flash 4.0 v2 sCMOS camera from Hamamatsu Photonics detects fluorescence and brightfield channels. The exposure time for each point in the Raman measurement is 1.2 s, and laser power at the sample plane is 100 mW. Each FOV is 50x50 pixels, 40x40 µm^2^, with each pixel corresponding to 800x800 nm^2^ in size. The time to acquire Raman hyperspectral images is 50 min per FOV. To conduct live-cell imaging, samples are incubated throughout the measurement using an onstage incubator to maintain physiological conditions. Raman hyperspectral maps are postprocessed via MATLAB (2021b) and RamApp, a web-based tool developed internally and publicly available (https://Ramapp.Io/; accessed on 6 July 2023), to correct for cosmic rays, detrend the signal baseline (fitted via adaptive smoothness partial least squares algorithm), smooth the signal along the wavelength axis (via Savitzky–Golay filtering). Each Raman hyperspectral map is normalized by its Frobenius norm. Spectra are imported in Origin (Pro) (Version 2022. OriginLab Corporation, Northampton, MA, USA) for further analysis procedures. Ahead of principal component analysis (PCA) on the mean cell, vacuole, and background spectra, the dataset was standardized to obtain a normal data distribution (mean = 0 and standard deviation = 1). Hence, PCA is run on the standardized dataset through the PCA for Spectroscopy Origin (Pro) add-on. Multinomial logistic regression (MLR) is performed on PC scores and subcellular components through the Origin (Pro) MLR built-in function.

### 2.5. Statistical Analysis

When performing MLR, β coefficients statistics is calculated through two-tailed t-test with a 0.05 significance level, to evaluate the rejection of the null hypothesis stating a null β value and select significant principal components (PCs) to distinguish cell, vacuole, and background spectra. On the other hand, statistics of ODT-computed indexes are obtained via a Mann–Whitney U nonparametric test, with a significance level of 0.05, to allow for a nonnormal distribution of data points.

## 3. Results

### 3.1. MCF7 Doxorubicin-Induced Senescent Cells Engulf Neighboring, Vital Cells

**Figure 1:**
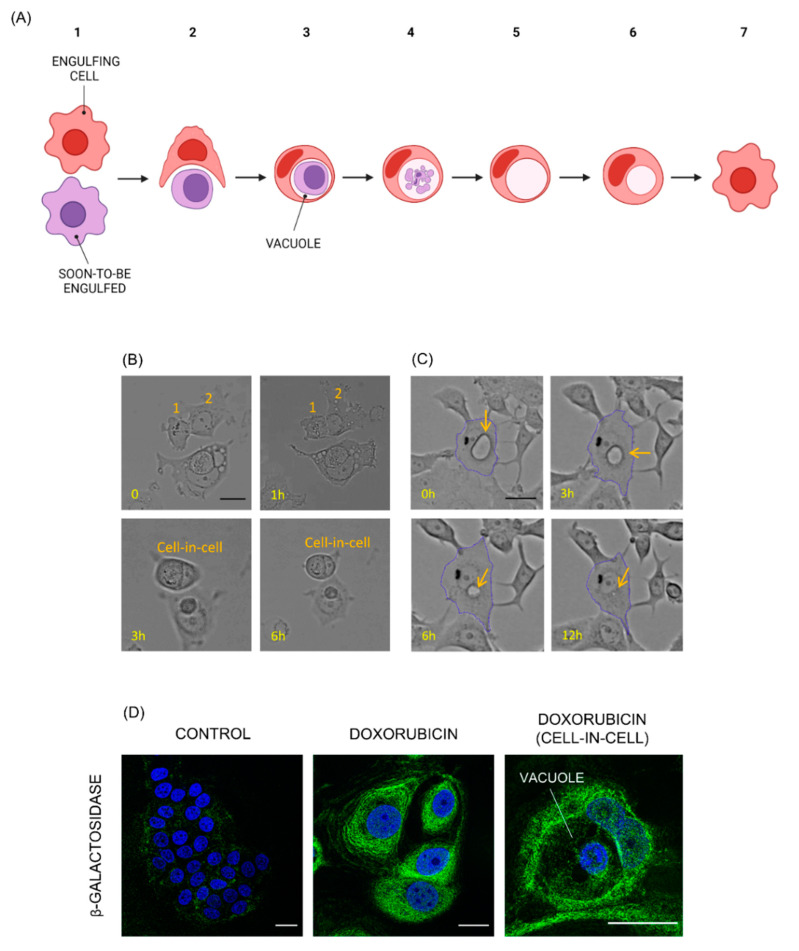
Doxorubicin-treated cancer senescent cells engulf and degrade neighboring ones. (**A**) Cartoon illustrating the engulfing process: a senescent cell engulfing a neighboring one (1, 2), originating a cell-in-cell structure (3). The engulfed cell is broken down in a vacuole within the engulfing one (4). After the degradation of the engulfing cell, the vacuole appears empty (5) and shrinks over time (6) until disappearing (7). Created with BioRender.com, accessed on 29 September 2023. (**B**) Timelapse image sequence of a doxorubicin-treated MCF7 cell (cell 1) engulfing a neighboring one (cell 2), leading to the creation of a cell-in-cell structure. Cells were treated with doxorubicin 48 h before starting the timelapse experiment. Scale bar: 25 μm. (**C**) Timelapse image sequence of a vacuole (indicated by the orange arrow) shrinking over time inside an MCF7 doxorubicin-treated cell. Cells were treated with doxorubicin 90 h before starting the time lapse experiment. Scale bar: 25 μm. (**D**) Control and doxorubicin-treated MCF7 cells stained for β-galactosidase activity (green) and nuclei (blue). In the last image, a cell-in-cell structure derived by the engulfing process. Scale bar: 25 μm.

To trigger the onset of senescence and its associated engulfing phenotype, we treated MCF7 cells with doxorubicin, a chemotherapeutic agent widely employed in antitumor clinical strategies across several tumor types, including breast [36,37]. After treatment with doxorubicin, surviving cells consistently engulfed and degraded neighboring ones. Engulfing cells constituted about 30% of the total cell population. The complete engulfing process is illustrated in Figure 1A. The internalization of a cell into another originated peculiar, round cell-in-cell structures (Figure 1B). The engulfed cells were broken down inside a big vacuole located in the cytoplasm of the engulfing ones over the span of 48 h. After degradation of the internalized cells, the vacuoles took up the most part of the cytoplasm of the engulfing ones and appeared empty when observed both in brightfield and in epifluorescence mode after staining with several classic fluorescent markers. After persisting for 12–15 h, vacuoles shrank over the span of 15 (additional) hours until disappearing, and the previously engulfing cells appeared whole and were not distinguishable anymore by those that did not engulf (Figure 1C).

Most MCF7 cells that were resistant to doxorubicin treatment were non-proliferative and displayed the polygonal, enlarged phenotype typical of senescence. However, to corroborate the establishment of the senescence state, control and treated cells were stained for β-galactosidase activity, the most accepted biomarker for senescence (Figure 1D). A dramatically increased positivity to the staining was observed as early as 48 h after treatment with doxorubicin. Cell-in-cell structures displayed markedly elevated β-galactosidase activity as well, indicating that engulfing cells were senescent. Notably, vacuoles appeared empty (black).

### 3.2. The Density of Vacuoles Measured by Optical Diffraction Tomography Is Comparable to That of the Aqueous Medium: Fixed Cell Imaging Using ODT

To precisely resolve the structure of cell-in-cells and vacuole-holding cells and investigate the vacuolar cargo, we applied ODT to MCF7 senescent engulfing cells. Tomocube’s ODT images the morphological structure of biological specimens under investigation by measuring RI values across their 3D volume in a label-free fashion [38]. Thus, ODT overcomes the obstacles and avoids the artifacts associated with fluorescence staining [12,13]. The ODT microscope here employed allows us to image live cells, but, because the structures we pointed to measure (cell-in-cells and vacuole-holding cells) were morphologically complex and part of a dynamic process, we decided to analyze PFA-fixed samples first. In fact, fixation stabilizes and protects cellular components and prevents them from moving, making them an easier target to image [39,40].

**Figure 2:**
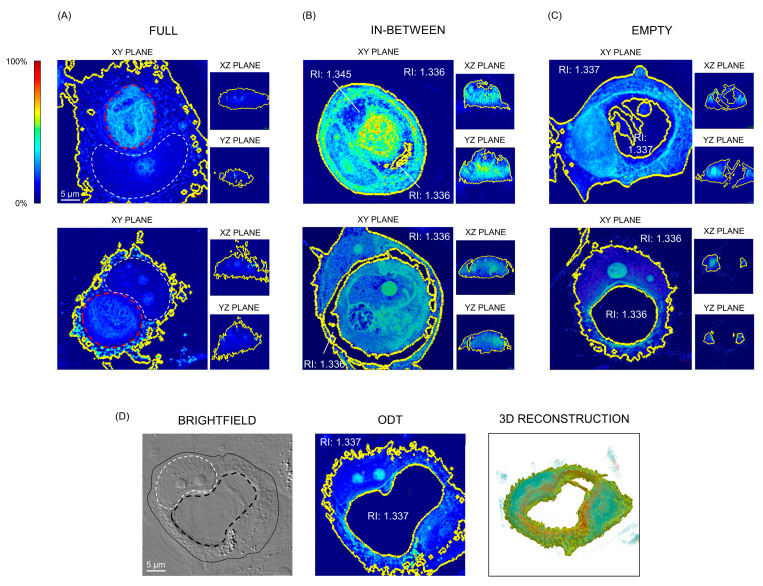
Representative images of PFA-fixed MCF7 cells using label-free cell tomography showing the main phases of the senescence-associated engulfment process. Each senescent engulfing cell undergoes these three stages in this order; two cells for each stage are shown. The yellow lines indicate the RI-based segmentation. (**A**) The engulfed cell (overlined with a discontinued red line) was clearly visible and took up all the space inside the cytoplasm of the engulfing cell, pressing its nucleus against the cell membrane. This caused the nucleus of the engulfing cell to acquire a halfmoon shape, indicated with a white discontinued line. All the cellular components presented a higher RI than the culture medium. (**B**) In this phase, the engulfed cell was being degraded within a vacuole in the engulfing one; it did not take up all the space in the engulfing cell cytoplasm anymore. Some regions of the vacuole presented the same RI as the culture medium. (**C**) The engulfed cell was not visible anymore, meaning it had been completely degraded. In the place of the engulfed cell was a hollow space, with a RI comparable to that of the aqueous medium. (**D**) Representative brightfield, ODT, and RI-based 3D reconstruction of a cell in the “empty” stage of the engulfing process. On the brightfield image, the white discontinued line outlines the nucleus of the engulfing cell, the black discontinued one indicates the vacuole, and the continue black line delineates the engulfing cell.

For clarity, we sorted the images of engulfing cells into three groups, based on the three main phases of the engulfment process: (i) a “full” situation, where the engulfing cell had just internalized another one and the vacuole was not visible (Figure 2A); (ii) an “in-between” situation, where the engulfed cell had not been entirely degraded yet, but the vacuole was already discernable (Figure 2B); and (iii) the “empty” phase, where the engulfed cell was not observable anymore, and the vacuole appeared completely empty when observed in brightfield (Figure 2C). We imaged 9 cells in the full stage, 3 in the in-between, and 16 in the empty one. As senescent cells are characterized by elevated intercell heterogeneity, segmentation was performed manually for each cell. As the cytoplasm is the least dense subcellular component, its minimum RI was set as the threshold to distinguish the cell from the aqueous medium (here constituted by PBS).

In most cases, the engulfed cell presented a higher mean RI than the engulfing, rendering it possible to distinguish the “inner” engulfed cell from the “outer” engulfing one solely on the base of their RI. In particular, the mean RI of engulfing cells, in all three phases of engulfment, was 1.347 ± 0.004, while the mean RI of engulfed ones was 1.364 ± 0.006. The RI values of the vacuoles (both in the in-between and in the empty phases) ranged from 1.333 to 1.340, which, for each cell, was comparable to the aqueous medium RI. Thus, it was not possible to distinguish between aqueous medium and vacuoles on the base of the RI. In fact, all cells in the empty stage of the engulfing process presented a crater within the cell itself when imaged through ODT (Figure 2C,D). As the RI of an object is directly correlated with its mass density, this result suggests that, after the degradation of the engulfed cells, vacuoles were in fact empty. This indicates that engulfing cells are capable of surviving for several hours (15–20 h) with a cavity that occupied a significant portion of the cellular volume, until shrinking and disappearance of the vacuole. In fact, the surface area of empty cells was, on average, higher than those of full and in-between cells (10,826, 8199, and 5603 µm^3^, respectively), but their volume/surface ratio was lower (1.280 vs. 1.521 and 1.941), reflecting their hollowness. Finally, the concentration and dry mass of full and in-between cells were higher than those of empty cells, unsurprisingly considering that engulfing cells in phases (i) and (ii) contained another, not yet degraded one.

### 3.3. Raman Spectroscopy Measures Show That Vacuoles Contain Low-Concentrated Biomolecules: Live Cell Imaging Using ODT and Raman

**Figure 3:**
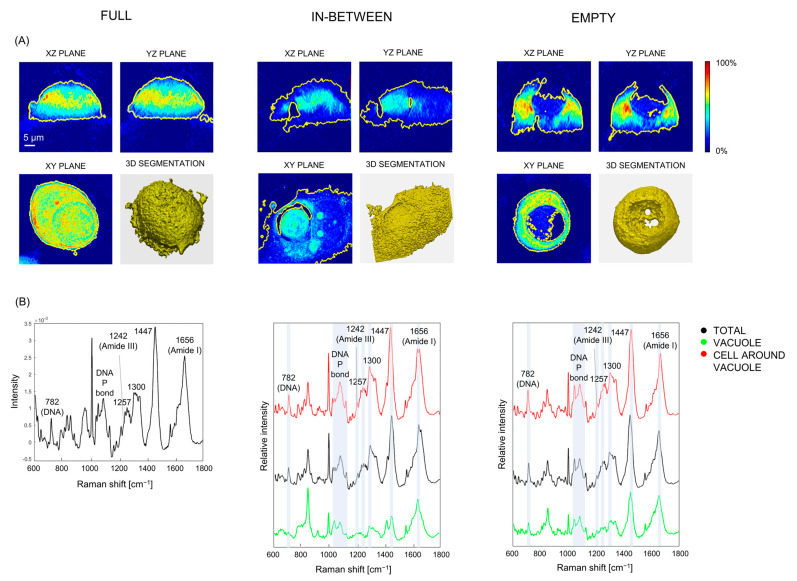
Representative ODT images (**A**) and Raman spectra (**B**) of vital MCF7 senescent cells in the three main stages of the engulfing process: full, with the engulfed cell still clearly visible; in between, with the engulfed cell being degraded within the engulfing one, and empty, with the vacuole inside the engulfing cell. When possible, for each cell measured with Raman spectroscopy (**B**), the average spectrum of the whole cell (including engulfed cell and vacuole when discernible, in black), the vacuole (green), and the engulfing cell surrounding the vacuole (red) were measured. The main Raman features are reported on the spectra (DNA P bond = DNA phosphodiester bond).

While fixation facilitates the precise measure of cell components by preserving cellular structures and locking them in place, it can also affect protein conformation and cause the loss of the soluble contents of the cells [41]. Thus, to confirm our initial findings, the ODT measures were repeated on live cells (Figure 3A), with results comparable to those obtained for fixed ones. Intrigued by the extremely low density of vacuoles, we applied Raman spectroscopy to engulfing cells in the three main stages of the engulfing process (Figure 3B). Raman enables the quantitative and in situ analysis of major classes of organic molecules. Thus, it was employed here to obtain the molecular composition and spatial organization of engulfing cells and especially vacuoles. Regardless of the engulfing phase, the total average spectra of engulfing cells, meaning that the sum of the spectrum of the engulfing cell, that of the engulfed one and/or that of the vacuole (displayed in Figure 3B in black), and the spectra of cells around the vacuoles only (shown in Figure 3B in red), reflected the typical Raman fingerprint of cancer cells, and in particular of MCF7, as reported in the literature [42,43]. The spectral analysis revealed Raman modes related to Amide I and III at 1656 and 1242 cm^−1^, respectively; a combination of proteins and lipids at 855, 1257, 1300, 1338, and 1447 cm^−1^; DNA at 782 cm^−1^; the DNA phosphodiester bond between 1010 and 1080 cm^−1^; and amino acids (including a relatively intense band at 855 cm^−1^, ascribable to tyrosine and further discussed in the following section). Interestingly, the Raman spectra of vacuoles (shown in Figure 3B in green) echoed those of cells, but with lower intensity: on average, the Raman intensity of vacuoles was half that of cells (the difference spectrum of cells and vacuoles is displayed in Supplementary Materials, Figure S1). This result indicates that, despite their extremely low RI registered using ODT, vacuoles were not entirely empty. In fact, the intensity of Raman modes scales linearly with the concentration of targeted molecular bonds in the irradiated volume. However, the low density of vacuoles was consistent with the lower intensity of Raman peaks in the vacuoles than in cells and, taken together and with their Raman profiles, these findings suggest that vacuoles contained macromolecules attributable to biological (cell-derived) material in extremely small concentration.

### 3.4. Vacuoles, Cells Surrounding the Vacuole and Aqueous Medium Can Be Distinguished on the Base of Their Raman Fingerprint

**Figure 4:**
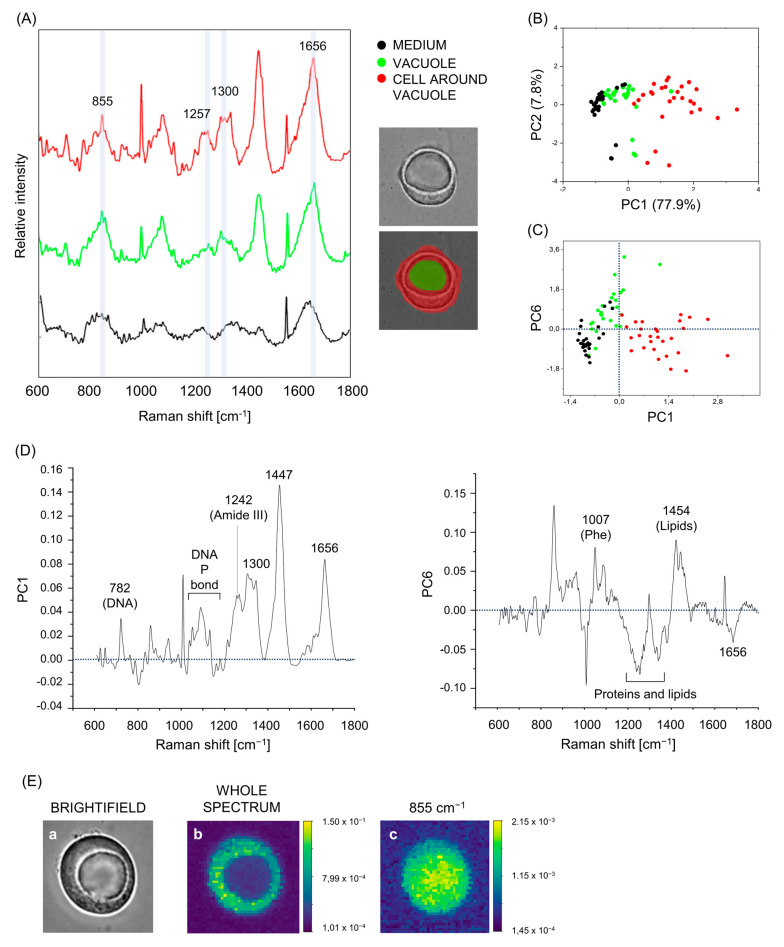
Spontaneous Raman can discriminate between cells, vacuoles, and culture medium. (**A**) Representative Raman average spectra of the cell around the vacuole (red), the vacuole (green), and the (culture) medium of a vital MCF7 engulfing cell. The main Raman features are reported on the spectra. Next to spectra, the brightfield picture of the cell. (**B**) PCA space showing Raman spectra of medium (black), vacuoles (green), and cells surrounding vacuoles (red). (**C**) Scatter matrix obtained by plotting the most significant PC scores (MLR coefficients featuring *p* < 0.01) for the separation of the three groups (cell around vacuole, vacuole, and background): PC1 and PC6. (**D**) Loading spectra of PC1 and PC6. The main Raman features are reported on the spectra (DNA P bond = DNA phosphodiester bond; Phe = phenylalanine). (**E**) Representative images of an engulfing cell imaged in brightfield mode (a) and in spontaneous Raman mode (b,c) by selecting the full spectral range (600–1800 cm^−1^), (b) or selecting the peak 855 (c).

As senescent cells are characterized by an extremely elevated secretion of mediators [44,45], Raman bands ascribable to biomolecules commonly found in cells could be observable in the spectra of the culture medium as well. To demonstrate irrevocably that, despite their low RI, vacuoles were not constituted of plain aqueous medium, their Raman profile should differ to that of the culture medium around the cells. To this end, and to investigate possible Raman peaks peculiar to vacuoles, we compared the spectrum of vacuoles not only to the cell surrounding it, but to that of the medium as well. To average cell-to-cell variability, Raman spectra of several cells holding empty-looking vacuoles were measured. All cells were imaged live, adherent to the bottom of a quartz Petri dish in the presence of RPMI. For each image, corresponding to one cell, the spectra of the medium, the vacuole, and the (engulfing) cell surrounding the vacuole were measured (Figure 4A), and a PCA was applied to the average spectra of these three groups from 28 measured cells. As shown in Figure 4B, the PCA could clearly separate the three groups (‘’medium’’, ‘’vacuole’’, and ‘’cell around the vacuole’’). Amongst the 20 PCs computed in the analysis, the PC1 and PC6 were retained for comparison between groups, as they showed the most significant values for regression coefficients in an MLR model using PC scores as independent variables and the medium, cell, and vacuole labels as dependent categorical variables (*p* < 0.01) (Figure 4C) and were particularly useful for the visualization of differential Raman features/intensities among the three groups. The PC1 score mainly represented the cell, while the PC6 score discriminated between medium and vacuoles, as vacuoles displayed positive PC6 scores whereas cells and medium showed negative PC6 scores (Figure 4C). Along the PC1 axis, representing the cell material, the spectra from the vacuoles disposed in the PCA space in-between the spectra of the medium and the cell (Figure 4B,C). This denotes an intermediate biochemical nature of the vacuole with respect to the cell, showing strongly positive PC1 scores, and the aqueous medium, showing strongly negative PC1 scores.

The Raman profile of the medium was overall different from that of vacuoles and cells, but, as expected, we found Raman features of biomolecules in the culture medium, in particular a peak at 1656 cm^−1^, ascribable to the vibration of the OH group in water but also to Amide I, and protein and lipids at 1300 and 1257 cm^−1^, consistently with the protein and lipid secretion carried out by senescent cells. Notably, the profile of PC6 (Figure 4D) indicates that vacuoles, compared to cells and medium, are abundant in lipids (1454 cm^−1^), but lack protein content (as indicated by the peaks at 1656 and 1007 cm^−1^, of Amide I and phenylalanine, respectively). The band at 1560 cm^−1^ is a system artifact caused by a hot pixel in the CCD.

In accordance with our previous results, vacuoles differed from cells especially in the relative concentration of the main components, not in their absence or presence of other molecular species. These results confirm that vacuoles contained typical cell-derived biomolecules but in low concentrations, consistent with the notion that engulfed cells are degraded within the vacuoles in engulfing ones. It is reasonable to hypothesize that the biomolecules found in vacuoles derived from broken-down cells previously engulfed, and, assumingly, this biological material was transferred out of the vacuoles to the engulfing cells overtime, concurrently with vacuole shrinking, providing the engulfing cells with nutrients and building blocks necessary for their persistence. This is in line with the results obtained with standard techniques in other laboratories [8,10,46]. Of note, Raman bands between 800 and 900 cm^−1^, and particularly a peak at 855 cm^−1^ (Figure 4E), together with a peak at 965, were found in vacuoles at relatively high concentrations, with peak intensities as high as or higher than in cells. Raman peaks at 837, 845, and 965 cm^−1^ are attributable to tyrosine [47], while 874 is a peak of tryptophan [48]. The peak at 855 cm^−1^ is ascribable to triglycerides, but also to tyrosine [48].

### 3.5. Vacuoles in Different Phases of the Engulfing Process Can Be Partially Separated on the Base of their Raman Spectra

**Figure 5:**
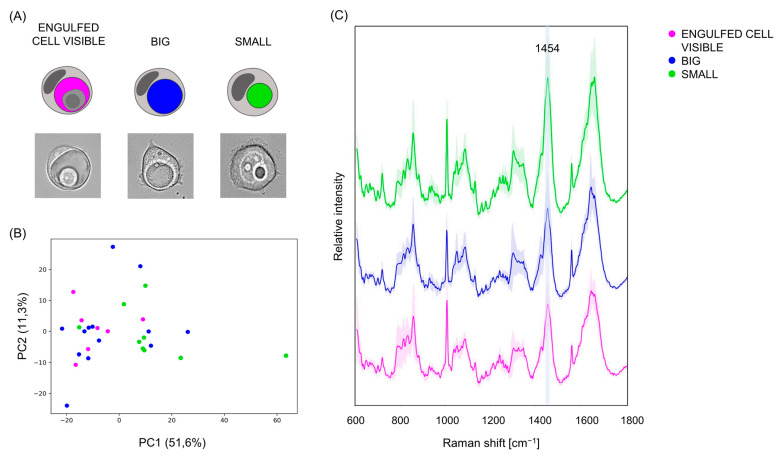
Spontaneous Raman to discriminate between vacuoles in different stages of the engulfing process. (**A**) Illustration (first row) and brightfield vacuole pictures (second row) showing their classification into the three groups (i) “engulfed cell visible”, (ii) “big”, and (iii) “small”. Created with BioRender.com. (**B**) PCA of Raman spectra of “engulfed cell visible” (pink), “big” (blue), and “small” (green) vacuoles. (**C**) Average spectra of “engulfed cell visible” (pink), “big” (blue), and “small” (green) vacuoles. Shaded regions display values within one standard deviation of the mean.

Finally, having demonstrated that vacuoles can be distinguished from the surrounding cells and from the background on the base of their Raman fingerprint, we moved on to dissect the heterogeneity among vacuoles. We sorted the previously imaged vacuole-holding cells into three groups (Figure 5A): (i) “engulfed cell visible”, for images where the engulfed cell was not entirely degraded yet, thus still visible inside the vacuoles; (ii) “big”, if the vacuole took up more than half the total area of the engulfing cell; and (iii) “small”, as opposed to “big”, for cases where the vacuole occupied less than half the total area of the cell. The PCA (Figure 5B) offered a partial separation. Once again, vacuoles in the three groups did not differ for the presence or absence of specific peaks, but we found a few Raman bands whose intensities differed visibly between the average spectra of the three groups (Figure 5C), especially the peak at 1454 cm^−1^ (Supplementary Materials, Figure S2), attributable to lipids. In particular, its intensity was higher in the group “small” than in the other two, possibly suggesting a relative accumulation of lipids as the vacuoles became smaller. This is in line with our previous results indicating that vacuoles were richer in lipids than the aqueous medium (see Section 3.4). The difference spectrum of vacuoles in the group “big” and vacuoles in the group “small” and the partial least squares discriminant analysis (PLS-DA) and most significant PCs is displayed in Supplementary Materials (Figures S3 and S4).

Overall, these data, although promising, are not sufficient for a clear separation of vacuoles based on their Raman fingerprint. However, the careful real-time monitoring of a greater number of cells might enable the detection of small but significant differences among vacuoles at different stages of the engulfing process.

## 4. Discussion

In this study, we employed ODT and confocal Raman microscopy to gain insight into the nature and content of vacuoles that generate inside therapy-resistant cancer senescent cells after engulfing and degradation of other vital, neighboring ones. This process has been observed in several tumors in vivo [10] and is correlated with poor prognosis, as it provides nutrients and building blocks to the engulfing cells, allowing for the persistence of deleterious cancer cells even in hostile environments [8,9]. By measuring the RI of vacuole-holding cells, we found that vacuole density was lower than that of the cytoplasm and comparable to the PBS or culture medium. Conversely, the chemical map of vacuoles obtained through Raman microscopy differed appreciably from that of the aqueous medium (PBS or RPMI). The Raman profile of vacuoles was qualitatively identical to that of the corresponding surrounding cell, but peak intensities in vacuoles were markedly lower. As peak intensity scales directly with the concentration of the corresponding chemical species, this result indicates that biomolecules were in small concentration within the vacuoles, consistent with the low mass density in vacuoles measured through ODT. Taken together, our results indicate that engulfing-originated vacuoles were not comparable to the medium despite their appearance; instead, they contained diluted biomolecules characteristics of cells, most likely derived from previously broken-down engulfed cells.

The extremely low RI of vacuoles may suggest that the breakdown of the engulfed cells within the vacuoles is not executed through enzymatic activity, as enzymatic degradation of different biomolecules requires the presence of several macromolecules/protein complexes; hence, it is reasonable to assume that vacuolar density and the intensity of Raman peaks of proteins would be higher. In the last decade, it was proposed that internalized cells as found in cell-in-cell structures that originated both after senescence induction [8] and from another, similar type of engulfment called entosis are killed by a mechanism dependent on acidified lysosomes [49,50]. In a recent study, Su and colleagues [11] showed how a remarkable decline in vacuolar pH determines the death of internalized cells following entosis in several breast cancer cell lines, and described vacuoles as “huge lysosomes”. It is reasonable to hypothesize that a similar mechanism applies to our model. Notably, senescent cells have an exceptionally expanded lysosomal compartment [51] and, while the inside of vacuoles was negative to several common fluorescent markers, we found that the vacuolar membrane was positive to the lysosome-targeting LysoTracker probe (Supplementary Materials, Figure S5). This indicates that vacuoles really are big lysosome-like structures inside which internalized cells are broken down and implies that the vacuole membranes effectively segregate the acid environment from the cytoplasm of the engulfing cell to prevent it from getting damaged. This, in turn, would explain in part why vacuoles appear empty even following different staining, as the vacuolar membrane might block the dyes out.

By statistically comparing the spectra of vacuoles with those of the cells surrounding them, we found that the two groups differed solely in peak intensities, and not in the presence or absence of specific Raman bands. The Raman intensity profile of vacuoles was half that of the cells, but with the notable exception of the Raman region between 800 and 900 cm^−1^ and the peak at 965, for which intensities were equally great or greater in vacuoles than in cells. Raman peaks at 837, 845, 855, and 965 cm^−1^ can be attributed to tyrosine [47], while the peak at 874 cm^−1^ is associated with tryptophan [48]. Interestingly, tyrosine and tryptophan are two of the three aromatic amino acids (AAAs, the third being phenylalanine), and elevated levels of AAAs have been found in breast cancers [47,52]. AAAs are critical intermediates that connect nucleotide, glucose, and lipid metabolism, and represent key precursor to many biological compounds involved in the regulation of many metabolic pathways and necessary for cellular functioning [47,52,53]. It should be taken into consideration that AAAs are more efficient Raman scatterers and thus are more easily detectable than many other biomolecules. This may be an explanation for why the intensities of AAA-derived Raman bands stayed high even in vacuoles. The other possible explanation is an actual relative accumulation of tyrosine and tryptophan inside vacuoles. As the content of vacuoles is supposedly transferred to the surrounding senescent engulfing cells, the relatively predominant presence of tyrosine and tryptophan within the vacuoles could reflect an elevated demand for these amino acids from engulfing cells to support their exceptionally elevated metabolism [54,55], leading to an inflated degradation of AAA-containing peptides to meet that demand. On the other hand, AAAs are involved in the production of reactive oxygen species, with deleterious effects on cells [53]. Thus, it is possible that tyrosine and tryptophan are kept within the confined space of vacuoles as long as possible, instead of being transferred quickly to the cell like the other biomolecules, to prevent them for damaging the cell itself, which would result in their relative accumulation in vacuoles. Finally, tyrosine and tryptophan are the biggest amino acids (181 and 204 Da, respectively), with great steric hindrance. The simplest explanation for their relative buildup inside the vacuoles might be that their size obstacles their transit through the vacuole membrane and into the engulfing cell cytoplasm.

In conclusion, in this work, we showed a feasibility of using ODT and Raman for monitoring cell engulfment process from a limited number of cells. We demonstrated how this approach can be greatly beneficial for describing and monitoring this and other complex biological processes in a noninvasive, label-free fashion, crucial for more effective biomedical research.

Further studies with an extended cell dataset will follow to confirm the mechanism underlying the degradation of internalized cells and to investigate the process through which the biomolecules derived from the breakdown of engulfed cells are transferred to the engulfing ones to be exploited as nutrients/building blocks. Although our existing custom-built Raman microscope allows for high-speed Raman imaging of live cells [35,56], it is still based on the point-scanning measurement. Obtaining data from a large number of cells or tissue slices is challenging. We are currently building a new Raman imaging system to overcome this limitation by exploiting the coherent Raman mechanism. This new system will allow for collecting Raman data from large number of live cells in a short period of time so that we can extend the study with statistical analysis. Future studies will also include timelapse monitoring of the overall process, instead of discrete snapshots as shown here: ideally, individual cells should be monitored in real time with both ODT and Raman microscopy, and the morphological and chemical information gathered over time for each cell combined for a better interpretation of the engulfing process.

## Data Availability

All data needed to evaluate conclusions in this paper are present in the paper, Supplementary Materials, and the Zenodo repository (https://zenodo.org/doi/10.5281/zenodo.8359788, accessed on 29 September 2023).

## Supplementary Materials

The following supporting information can be downloaded at: https://www.mdpi.com/article/10.3390/bios13110973/s1, Figure S1: Average difference spectrum obtained from the subtraction of the spectrum of each “cell around vacuole” and the corresponding vacuole; Figure S2: Boxplot showing the intensity at the 1454 cm⁻¹ peak in the average Raman spectra of “engulfed cell visible” (pink), “big” (blue), and “small” (green) vacuoles; Figure S3: Spectrum obtained from the subtraction of the mean spectrum of vacuoles in the group “big” and vacuoles in the group “small”; Figure S4: Display of the partial least squares discriminant analysis (PLS-DA) and most significant principal components (PCs) of vacuoles in different stages of the engulfment process; Figure S5: Representative images of senescent MCF7 vacuole-holding cells stained for lysosomes with LysoTracker.
