# Supplementary material for: Optical Diffraction Tomography and Raman Confocal Microscopy for the Investigation of Vacuoles Associated with Cancer Senescent Engulfing Cells
Source: Biosensors (Basel). 2023 Nov 7;13(11):973. doi: 10.3390/bios13110973 (PMC10669708; doi:10.3390/bios13110973)
Supplement: Supplementary file 1 [file biosensors-13-00973-s001.zip › biosensors-2667782-supplementary.pdf]

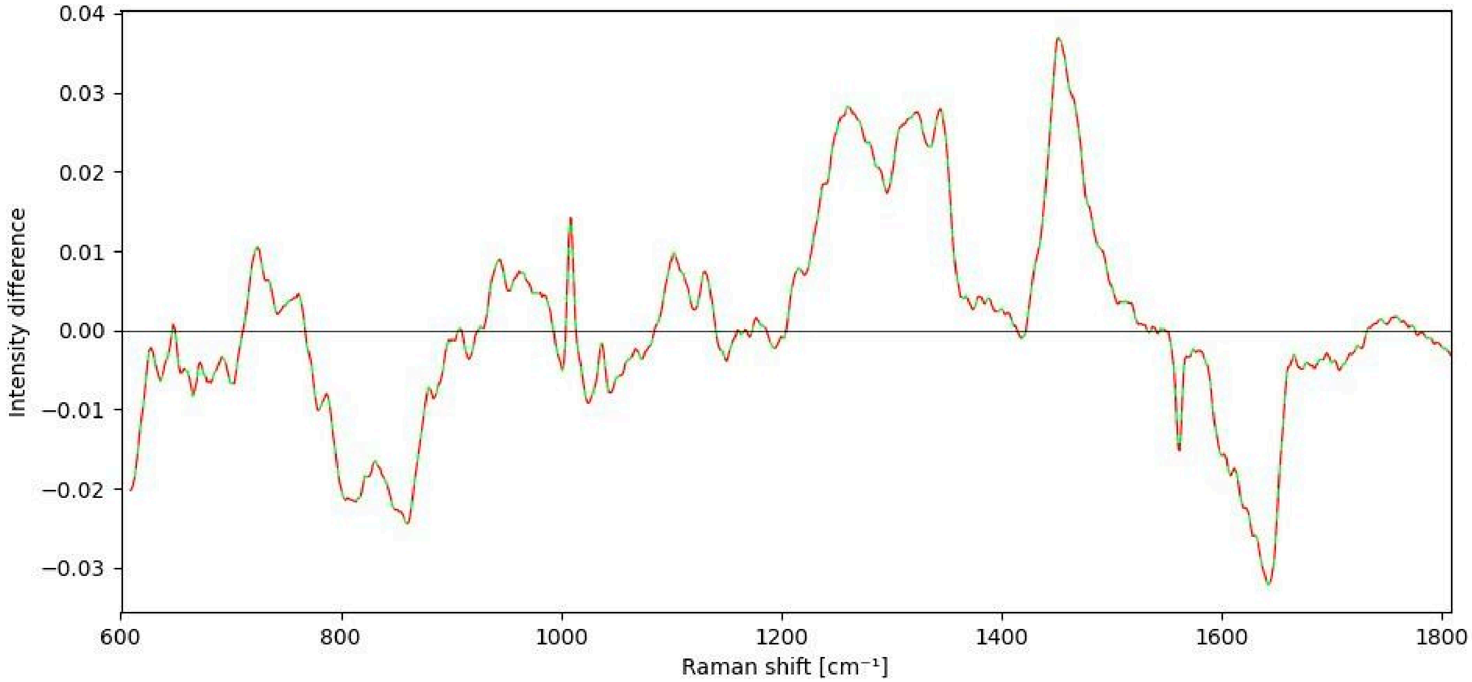

Figure S1. Average difference spectrum obtained from the subtraction of the spectrum of each *cell around vacuole* and the corresponding vacuole. Spectra were first normalized to unit vector (L2 norm).

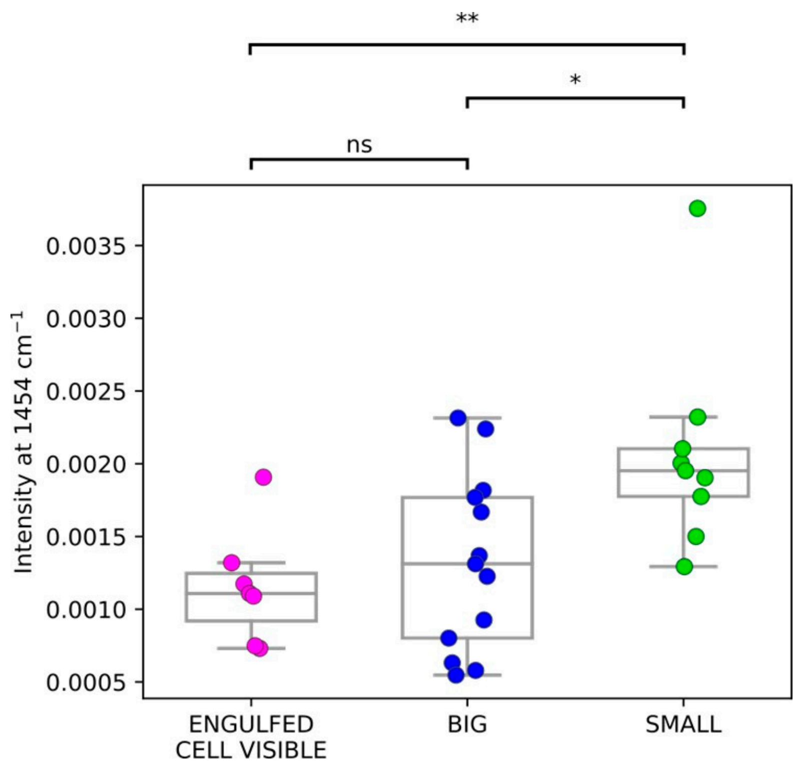

Figure S2. Box plot showing the intensity at the 1454 cm^{-1} peak in the average Raman spectra of *engulfed cell visible* (pink), *big* (blue), and *small* (green) vacuoles. The Mann-Whitney U test shows significant differences in the intensity between *engulfed cell visible* and small ($p<0.01$) and between *big* and *small* ($p<0.05$).

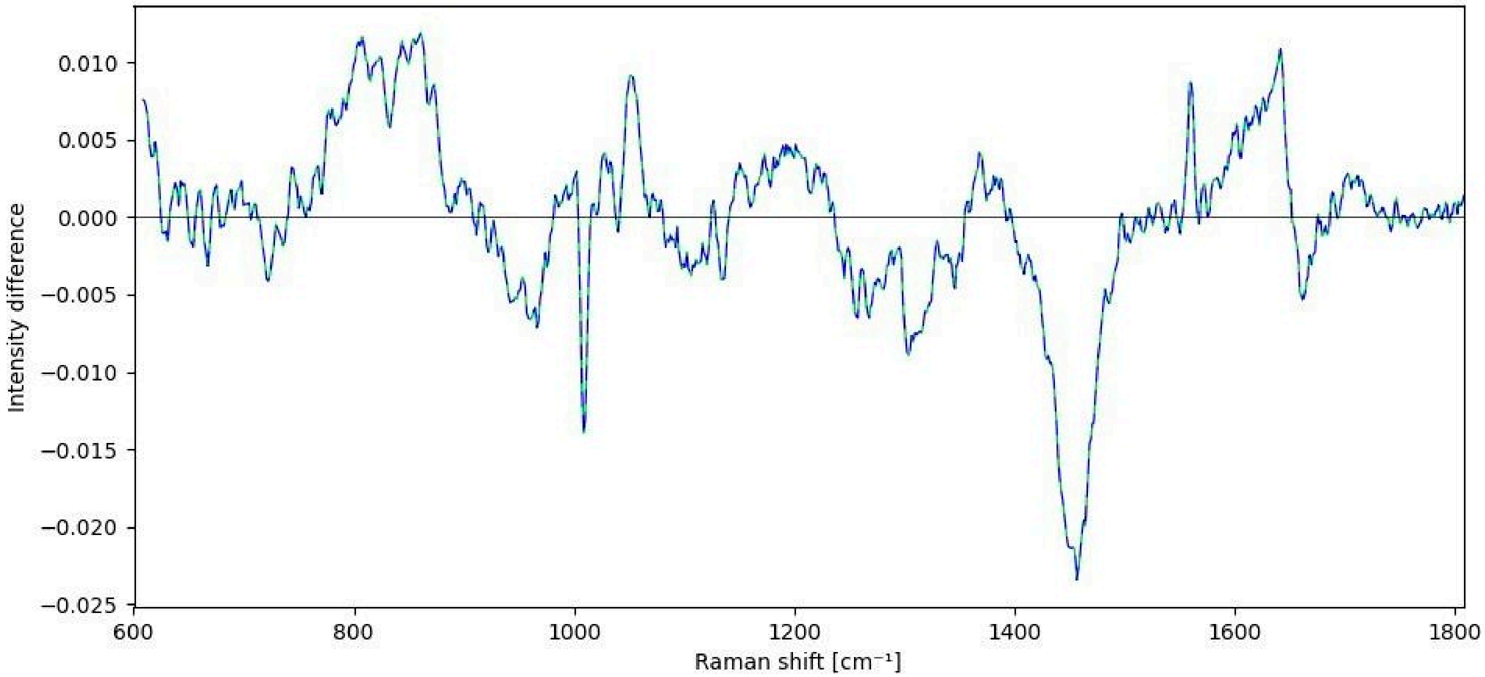

Figure S3. Spectrum obtained from the subtraction of the mean spectrum of vacuoles in the group *big* and vacuoles in the group *small*. Spectra were first normalized to unit vector (L2 norm).

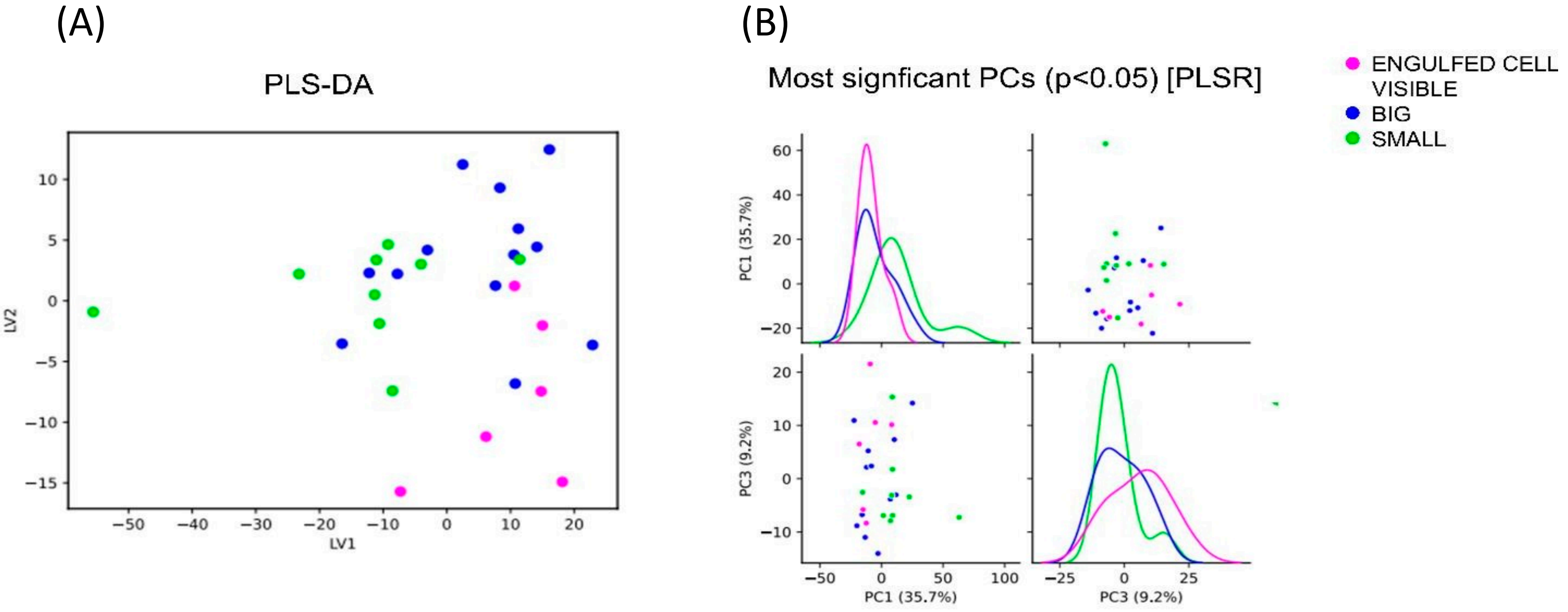

Figure S4. Display of the partial least squares discriminant analysis (PLS-DA) **(A)** and most significant principal components (PCs) **(B)** of vacuoles in different stages of the engulfment process (*engulfed cell visible* in pink, *big* in blue and *small* in green). For the PLS-DA, the mean spectra of vacuoles were imported into Python and standardized as described in the *Material and Methods* section; the PLS-DA was performed using the one-hot-encoded stage as the response variable.

(A) (B)

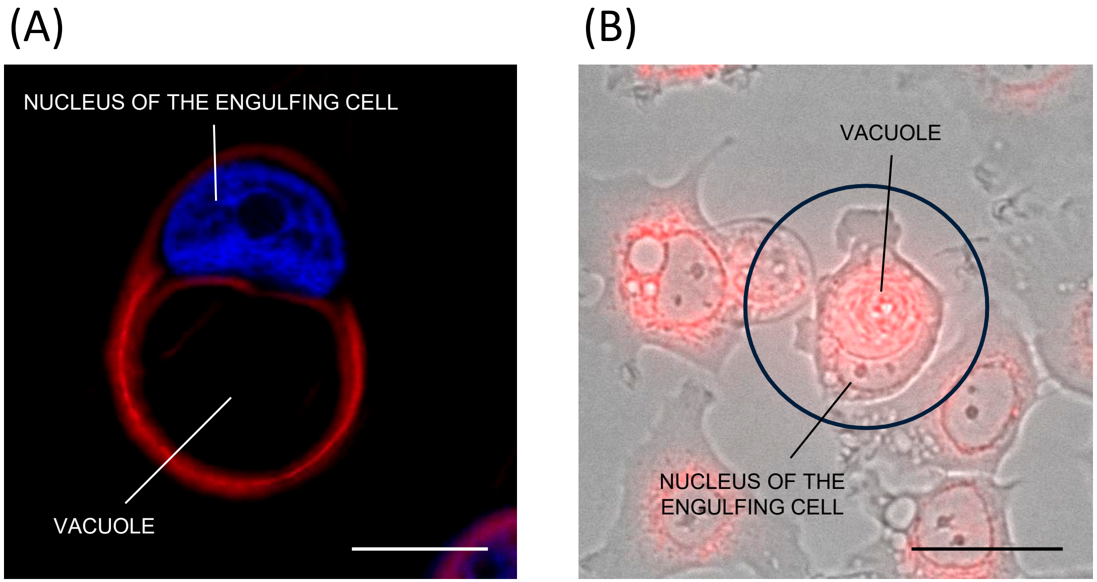

Figure S5. Representative images of senescent MCF7 vacuole-holding cells stained for lysosomes with Lysotracker. The picture **(A)** was obtained with a confocal microscope in fluorescent mode. In blue (DAPI staining) is the engulfing cell nucleus. Scale bar: 25 µm. The picture **(B)** was obtained in brightfield mode. The circle indicates the engulfing cell. Scale bar: 50 µm.
